# Determination of qPCR reference genes suitable for normalizing gene expression in a novel model of Duchenne muscular dystrophy, the D2-*mdx* mouse

**DOI:** 10.1371/journal.pone.0310714

**Published:** 2024-11-13

**Authors:** Brigida Boccanegra, Roberta Lenti, Paola Mantuano, Elena Conte, Lisamaura Tulimiero, Richard J. Piercy, Ornella Cappellari, John C. W. Hildyard, Annamaria De Luca

**Affiliations:** 1 Department of Pharmacy-Drug Sciences, University of Bari “Aldo Moro”, Bari, Italy; 2 Department of Clinical Sciences and Services, Comparative Neuromuscular Diseases Laboratory, Royal Veterinary College, London, United Kingdom; University of Minnesota Medical School, UNITED STATES OF AMERICA

## Abstract

Duchenne muscular dystrophy (DMD) is a X-linked neuromuscular disorder arising from mutations in the dystrophin gene, leading to a progressive muscle wasting and disability. Currently there is no universal therapy, and there is thus a strong interest in preclinical studies for finding novel treatments. The most widely used and characterized mouse model for DMD is the C57BL/10ScSn-*Dmd*^*mdx*^*/*J (BL10-*mdx*), but this model exhibits mild pathology and does not replicate key features of human disease. The D2.B10-*Dmd*^*mdx*^/J (D2-*mdx*) mouse is a more recent model which seems to better mimics the complex human DMD phenotype. However, the D2-*mdx* mouse remains less extensively characterised than its BL10-*mdx* counterpart. Quantitative PCR analysis of gene expression is an important tool to monitor disease progression and evaluate therapeutic efficacy, but measurements must be normalised to stably expressed reference genes, which should ideally be determined and validated empirically. We examined gene expression in the gastrocnemius (GC), diaphragm (DIA) and heart in the D2-*md*x mouse, the BL10-*mdx* mouse, and appropriate strain-matched wild-type controls (D2-wt and BL10-wt), from 4 to 52 weeks of age, using a large panel of candidate references (*ACTB*, *AP3D1*, *CSNK2A2*, *GAPDH*, *HPRT1*, *PAK1IP1*, *RPL13A*, *SDHA*, and in the heart, also *HTATSF1* and *HMBS*). Data was analyzed using GeNorm, Bestkeeper, deltaCt and Normfinder algorithms to identify stable references under multiple possible scenarios. We show that *CSNK2A2*, *AP3D1* and *ACTB* represent strong universal reference genes in both GC and DIA, regardless of age, muscle type, strain and genotype, while *HTATSF1* and *SDHA* are optimal for the heart. *GAPDH*, *HPRT1* and *RPL13A* were conversely revealed to be poor references, showing tissue-, age- or disease-specific changes in expression. Our results illustrate the importance of determining appropriate reference genes for specific comparative scenarios, but also reconfirm that universal panels can nevertheless be identified for normalising gene expression studies in even complex pathological states.

## 1. Introduction

Duchenne muscular dystrophy (DMD) is a rare paediatric X-linked neuromuscular disorder. The pathology arises from mutations in the dystrophin gene, leading to the absence of dystrophin, a sub-sarcolemmal protein which is part of the dystrophin glycoprotein complex (DGC) [1]. Dystrophin and DGC have both structural and signalling roles, providing a mechanical link between the intracellular cytoskeleton and the extracellular matrix, thus allowing proper muscle contraction and a mechano-metabolic coupling [1]. In the absence of dystrophin, this mechanical link is lost, the DGC complex fails to assemble and muscle fibres become vulnerable to contraction-induced injury, resulting in repeated cycles of degeneration and compensatory regeneration [2]. Ultimately, exhaustion of satellite cells [the muscle resident stem cells] and/or their fibroadipogenic transition occurs, contributing to failing regeneration and progressive fibrosis [2, 3]. This process is associated with, and exacerbated by, a complex pathogenetic cascade, including persistent inflammation, oxidative stress and dysmetabolism, with multiple events being primarily and secondarily modulated in the pathology [3, 4]. DMD presently has no cure, with the gold standard of treatment being palliative corticosteroids: these delay pathological progression without addressing the underlying defect. There are several therapeutic approaches now approved or in clinical trials, including gene therapy or antisense oligonucleotides (ASO) for exon skipping (to restore an internally truncated dystrophin form, converting DMD to the milder Becker muscular dystrophy, BMD) [5]. However, therapies based on a mutation-specific approach such as exon skipping are therefore suitable only for a subset of patients. Several drugs (newly synthesised or repurposed) are in preclinical or clinical trial [6] with the aim of targeting different aspects of the complex pathogenetic cascade resulting from the absence of dystrophin.

Animal models are essential for evaluating potential therapeutics, and several such models are available for DMD preclinical research, ranging from simpler (i.e., *Sapje* zebrafish) to more complex, such as dystrophic dogs and pigs [7–12]. The most widely used animal model is the C57BL/10ScSn-*Dmd*^*mdx*^*/*J: the BL10-*mdx*, or simply ‘*mdx’* mouse [13], which lacks dystrophin due to a premature termination codon (PTC) in exon 23. This exon lies in-frame and skipping of exon 23 thus restores dystrophin protein: this model is consequently of value for assessing therapeutic dystrophin restoration and functional improvement [14]. The *mdx* mouse does, however, present a markedly milder phenotype and a slower disease progression than is seen in human patients, with compensatory muscle hypertrophy rather than atrophy, and with limited muscle fibrosis, mostly observed in the diaphragm: these features limit the utility of this animal model. The D2.B10-Dmd^*mdx*^/J (D2-*mdx*) mouse is a more recent murine model, generated by crossing the classic BL10-*mdx* with DBA/2J wild type (D2-wt) mice [15, 16]. This model appears to more closely mimic human pathology, exhibiting a more severe pro-fibrotic and pro-inflammatory phenotype due to polymorphisms in the Latent Transforming Growth Factor Beta Binding Protein 4—*LTBP4* gene [17–19], a Transforming Growth Factor Beta 1—TGF-β key regulator, also detected in DMD patients [7, 8]. Moreover, skeletal and cardiac muscles of D2-*mdx* mice exhibit calcifications, due to the presence of the *Dyscalc* loci in the ATP Binding Cassette Subfamily C Member 6—*ABCC6* and Epithelial Membrane Protein 3—*EMP3* genes [16, 20]. This, in addition to the impaired self-renewal capacity [15] due to an alternative splicing of the Annexin A6—*ANXA6* gene, overall contributes to a faster progression of disease and early cardiomyopathy [16]. As a rather new animal model of DMD, the D2-*mdx* mouse lacks the historical pedigree of the classic BL10-*mdx*: to address this, a characterisation of disease progression and severity *via* a common project of natural history study is carried on independently in our laboratory and in the Leiden University Medical Center, LUMC (Prof. Annemieke Aartsma-Rus’ lab). Here we further expand this characterisation by identifying suitable stable reference genes for use in gene expression studies using this new model, an approach that is currently independently ongoing in LUMC’s laboratories.

Quantitative measurement of gene expression via qRT-PCR is crucial for investigating transcriptional changes associated with physiological conditions (e.g., aging) and diseases. Pharmacologically, qRT-PCR is also crucial for drug testing to assess efficiency/efficacy of a specific therapy by measuring modulation of target genes. This is particularly important for rare diseases as DMD, allowing insights into the molecular mechanisms governing disease progression, pinpointing potential therapeutic targets and designing tailored interventions that can potentially modify or alleviate the course of the disease [21–23].

To obtain meaningful quantitative gene expression data, it is essential that measurements are first normalised to internal, stably expressed ‘reference genes’ (also known as housekeeping genes). Identification of such reference genes can be challenging, however, as it is increasingly clear that no “universal” reference gene exists even those used historically (typically *18S*, *GAPDH* and *ACTB*) can vary under some conditions, and thus appropriate references should be determined for each new comparative scenario. It is moreover well established that the use of a single reference gene is not sufficient [24], therefore it is advantageous to use a large candidate reference gene panel to allow identification of several genes appropriate for the experimental settings considered. As previously published [25, 26], with animal models even different muscles of the same animal might need different reference genes. This is of particular relevance in dystrophic models: while healthy skeletal muscle is transcriptionally comparatively stable, dystrophic muscles show a dynamic and highly variable setting. The complexity of the pathophysiological cascade triggered by dystrophin absence involves many different cell types, multiple cycles of degeneration and regeneration and complex interplay between inflammation and fibrosis, making identification of stable reference genes complicated. Nevertheless, previous work demonstrated the viability of this approach, establishing appropriate reference genes for the *mdx* mouse model [25]. Since the D2-*mdx* represents an emergent but not yet comprehensively characterized mouse model, here we complement these earlier studies by extending them to this new model. Using the D2-*mdx* mouse, its wild-type counterpart (D2-wt), and the original BL10-*mdx* mouse (and its WT counterpart, the BL10-wt), we searched the best candidate reference genes across two main skeletal muscles: the limb gastrocnemius (GC) muscle largely used for histology and molecular biology studies, and the more severely affected diaphragm (DIA), which is of particular clinical interest as it is one of the dystrophic mouse muscles that mirror pathological features found in human patients, such as fibrosis. In addition, we investigated expression within the heart, useful for translational studies since cardiomyopathy is a typical feature of DMD. Our analysis is comprehensive, spanning clinically-relevant ages throughout the mouse lifespan (4, 8, 12, 28, 52 weeks of age), and using a candidate panel of 8 reference genes for skeletal muscles screened from previously published work in the classical BL10-*mdx* (*ACTB*, *AP3D1*, *CSNK2A2*, *GAPDH*, *HPRT1*, *PAK1IP1*, *RPL13A*, *SDHA*), and 10 references for heart (all the above, with addition of the heart-specific candidates *HMBS* and *HTATSF1*). Our results provide a solid basis for further comparison of best reference genes in the mdx mouse models within the results obtained with the same approach in LUMC, so to strengthen the qPCR data on the exploratory genes of interest in complex disease models and overall, the need of robust standardized approaches.

## 2. Materials and methods

All the experiments were conducted in conformity with the Italian Guidelines for Care and Use of Laboratory Animals (D.L.116/92) and with the European Directive (2010/63/UE). The study was approved by the National Ethics Committee for Research Animal Welfare of the Italian Ministry of Health (authorization no. 513/2020-PR). Animal studies have been reported in compliance with the ARRIVE guidelines.

### 2.1 Animal groups and tissue collection

For this study male dystrophic mice of C57BL/10ScSn-*Dmd*^*mdx*^/J (BL10-*mdx*) and D2.B10-*Dmd*^*mdx*^/J (D2-*mdx*) backgrounds were used, as well as background-related wild-type mice of the C57BL/10SnJ (BL10-wt) and of DBA/2J (D2-wt). Mice were purchased from Jackson Laboratory (USA, distributed by Charles River, Calco, Italy). All mice were acclimatized in the animal facility before starting experimental procedures. Animals were housed in suitable cages (3–5 mice per cage), in a room where appropriate conditions of temperature (22–24°C), humidity (50–60%), and light/dark cycle (12 h/12 h) were constantly maintained for the entire duration of the study.

At the appropriate ages [4, 8, 12, 28, 52 weeks], all mice from each group were anesthetized *via* intraperitoneal [i.p.] injection with a cocktail of ketamine [100 mg/kg] and xylazine [16 mg/kg] and sacrificed by cervical dislocation; then, organs as well as muscles were harvested for postmortem analysis. For the purpose of this study, GC muscle, DIA, and heart of three animals from each group of all ages were collected and were embedded in a small amount of Tissue-Tek O.C.T. (Bio-Optica, Milan, Italy), immersed for 30s—60 s in isopentane cooled with liquid nitrogen (N2), and then stored at −80°C. This design ensures robustness, according to previous analytic approaches [25].

### 2.2 RNA extraction

Total RNA was isolated from unmounted tissue cryosections collected alongside those used for histology (100–150 sections of 10 μm, approx. 150-200mg tissue): sections were collected into pre-chilled microcentrifuge tubes and stored at -80°C until use. Room temperature lysis buffer (200 μL) from miRVana kit (cat. No. A27828, ThermoFisher Scientific) containing β-mercaptoethanol (1.4 μL) was then added directly to the samples. Solution and tissue were mixed through pipetting to ensure proper lysis. Extraction was performed with KingFisher™ Duo Prime (ThermoFisher Scientific) according to manufacturer’s protocols. RNA purity and quantity was assessed via spectrometry (ND-1000 NanoDrop, ThermoFisher Scientific), and only samples that showed 260/280 ratios >2.0 and 260/230 ratios >1.8 were considered acceptable.

### 2.3 cDNA synthesis

400 ng of purified RNA were reverse transcribed to cDNA with iScript gDNA Clear cDNA Synthesis Kit (172–5035 Bio-Rad Laboratories, Inc., CA, USA). To each RNA sample, 2 μl of Dnase master mix (volume by reaction: iScript Dnase 0.5 μL, iScript Dnase Buffer 1.5 μL) and nuclease-free water for a total reaction volume of 16 μl were added. The reaction was incubated using a thermal cycler at conditions 25° for 5 min followed by 75° for 5 min to inactivate DNAase. 4 μL of iScript Reverse Transcription Supermix 5X were then added, with the reaction incubated at 25° for 5 min, followed by 46° for 20 min with a final heat inactivation of 95° for 1 min, according to the protocol provided by the manufacturer [27, 28]. 80 μl of RNAse-free water were then added to the sample in order to obtain a 4ng/μl cDNA solution.

### 2.4 Real time PCR

Quantitative Real Time PCR was performed via the CFX384 Connect Real-Time PCR system (Bio-Rad, Hercules, CA, USA) using Prime PCR assays SYBR^®^ green. Each reaction used 2 μl of cDNA (8 ng cDNA, assuming 1:1 conversion), added with 5 μl of SssoAdvanced Universal SYBR^®^ Green Supermix (172–5271 Bio-Rad Laboratories, Inc., CA, USA), 0.5 μl of primers and 2.5 μl of nuclease-free water for a total volume of 10 μl.

All primer sequences for candidate reference genes are proprietary property of Bio-Rad Laboratories (Table 1) -anchor nucleotides and contexts lengths are available from the manufacturers.

**Table 1:** Primers of candidate genes purchased by Bio-Rad Laboratories.

Gene symbol	Gene full name	Unique assay ID
*ACTB*	Actin Beta	qMmuCED0027505
*AP3D1*	Adaptor Related Protein Complex 3 Subunit Delta 1	qMmuCID0022103
*CSNK2A2*	Casein Kinase 2 Alpha 2	qMmuCID0026331
*GAPDH*	Glyceraldehyde-3-phosphate dehydrogenase	qMmuCED0027497
*HMBS*	Hydroxymethylbilane Synthase	qMmuCID0022816
*HPRT*	Hypoxanthine Phosphoribosyltransferase 1	qMmuCID0005679
*HTATSF1*	HIV-1 Tat Specific Factor	qMmuCID0013600
*PAK1IP1*	PAK1 Interacting Protein 1/PIP1/WDR84	qMmuCID0021723
*RPL13A*	Ribosomal Protein L13a	qMmuCED0040629
*SDHA*	Succinate Dehydrogenase Complex Flavoprotein Subunit A	qMmuCID0019353

All qPCR performed using SYBR Green used a hot start (95°C for 2 min) followed by 40 cycles of a two-step reaction (95°C for 5 s, 60°C for 30 s). For each experiment, samples were analyzed in technical duplicate. The raw data were subjected to preliminary screening, and only technical duplicates with a standard deviation value <0.8 were considered acceptable for the analysis.

### 2.5 Analysis

Four distinct but complementary methods were used to assess the dataset: GeNorm, Bestkeeper, deltaCt and Normfinder. Each method employs a different approach for determining optimal reference genes, and thus genes ranked highly by all four are likely to be strong candidates (Table 2).

**Table 2:** Methodological approach of GeNorm, Bestkeeper, DeltaCt, NormFinder.

METHOD	APPROACH	DATA OUTPUT	INTERPRETATION OF RESULTS
*GeNorm*	Iterative pairwise approach.	M value: average of the pairwise variation of each gene with all other candidate genes.	Lower M values reflect high gene stability.
*Bestkeeper*	Pairwise comparison of each gene against the geometric average of all genes.	Pearson correlation coefficient.	R = 1 indicates perfect correlation
*DeltaCt*	Differences between Cq values for two genes within a given sample.	Standard deviation (SD) of deltaCt values.	Genes with low SD represent strong reference candidates
*Normfinder*	Assessment of absolute expression stability, either within the whole dataset or within and between subgroups.	Stability values (M).	Low values of M indicate strong references.

In detail:

- *GeNorm*: the GeNorm algorithm uses an iterative pairwise approach, determining the M value, the average of the pairwise variation of each gene with all other candidate genes. The software ranks genes based on the M value: the gene with the highest M value is then discarded and the process repeated until only a single pair of highly correlated genes remains, the ‘best pair’. Genes from unrelated functional categories that nevertheless exhibit a closely correlated expression profile across the dataset are thus assumed to reflect cDNA content within those samples.
- *Bestkeeper*: the Bestkeeper approach calculates the per-sample mean expression data of all candidate genes, creating a single consensus expression profile for the entire dataset: the ‘Bestkeeper’ (with the assumption that this multi-gene consensus approximates the overall cDNA content between samples). Individual genes are then compared with this consensus profile and ranked by their Pearson correlation coefficients, essentially determining which individual gene most closely mirrors the overall behaviour of all candidate genes.
- *DeltaCt*: The deltaCt is the difference between quantification Cq values (the number of cycles at which the fluorescence signal first crosses the threshold level) for two genes within a given sample: a pair of genes that vary in a consistent manner should exhibit concomitantly consistent deltaCt values across multiple samples, regardless of overall expression. By evaluating the standard deviation of deltaCt values across the dataset for each gene vs each other gene, the deltaCt method thus identifies genes which are consistently least variable with respect to all other genes: these represent strong reference candidates.
- *Normfinder*: the normfinder method ranks candidate reference genes by expression stability, where low stability values reflect minimal variation between samples (i.e., indicate strong references). The method can be used ‘ungrouped’, assessing overall variance of expression across the dataset (or subsets, as above),but can also carry out ‘grouped’ analysis, assessing variance of genes both within and between user-defined sample groups. This latter analysis is powerful, and can readily identify genes that are ostensibly stable, but nevertheless modestly but consistently up- or downregulated between such groups. Grouped analysis also generates a "best pair": two genes that can be combined to produce greater stability than any single gene alone [note: this is not equivalent to the best pair generated by geNorm]
- For this analysis, datasets were grouped (where applicable) by muscle type, healthy/dystrophic, strain or age.

With multiple muscles collected from multiple strains at multiple ages, our entire dataset comprised 180 samples. The breadth of this dataset thus allowed analysis to be further conducted on subsets: defined sub-categories of the full dataset such as ‘Diaphragm only’, or ‘dystrophic samples only’. This strategy (that we have employed previously [25, 26]) allows candidates ranked highly in the overall (full dataset) analysis to be evaluated in more specific contexts, and moreover can reveal candidates that score highly in one subset but not in another, potentially indicative of tissue or disease-specific behaviour.

For this study, the following subsets were assessed: disease-specific (healthy, dystrophic: each N = 90), muscle-specific (GC, DIA, heart: N = 60), strain-specific (BL10-wt, BL10-mdx, D2-wt, D2-mdx: N = 45) and age-specific (4, 8, 12, 28 and 52 weeks: N = 36) (Table 3).

**Table 3:** Experimental groups and number of samples analyzed.

	4 WKS	8 WKS	12 WKS	28 WKS	52 WKS	TOT
**BL10-wt**	3	3	3	3	3	**15**
**BL10-mdx**	3	3	3	3	3	**15**
**D2-wt**	3	3	3	3	3	**15**
**D2-mdx**	3	3	3	3	3	**15**
**TOT**	**12**	**12**	**12**	**12**	**12**	**60**

Number of samples analyzed:

15 samples for each strain multiplied for 3 muscles (GC, DIA, heart) = 45 samples *per* strain;

30 healthy samples multiplied for 3 muscles (GC, DIA, heart) = 90 healthy samples;

30 dystrophic samples multiplied for 3 muscles (GC, DIA, heart) = 90 dystrophic samples;

12 samples for each age multiplied for 3 muscles (GC, DIA, heart) = 36 samples *per* age;

60 total samples *per* muscle multiplied for 3 muscles (GC, DIA, heart) = 180 total samples analyzed.

As the validity of such analysis decreases when diminishing dataset size, further sub-sampling was not conducted. For the heart-specific dataset, however, analysis was repeated with inclusion of the two additional heart-specific gene candidates (*HTATSF1* and *HMBS*).

To integrate these four approaches and to generate an aggregate ranking, we further combined each output to generate consensus scores (geometric mean). The outputs of each algorithm are not directly equivalent: for Bestkeeper, high values indicate strong references, whereas for other approaches low values are indicative of strong candidates, and furthermore, some outputs are strictly clamped from 0–1, while others may exceed 1. To integrate all outputs, data was transformed accordingly: Bestkeeper data was first inverted (1-value), and then all scores were normalised to the highest value, placing all on a consistent scale and allowing per-gene geometric means to be derived [25].

## 3. Results

### 3.1 Cq data

**Fig 1:**
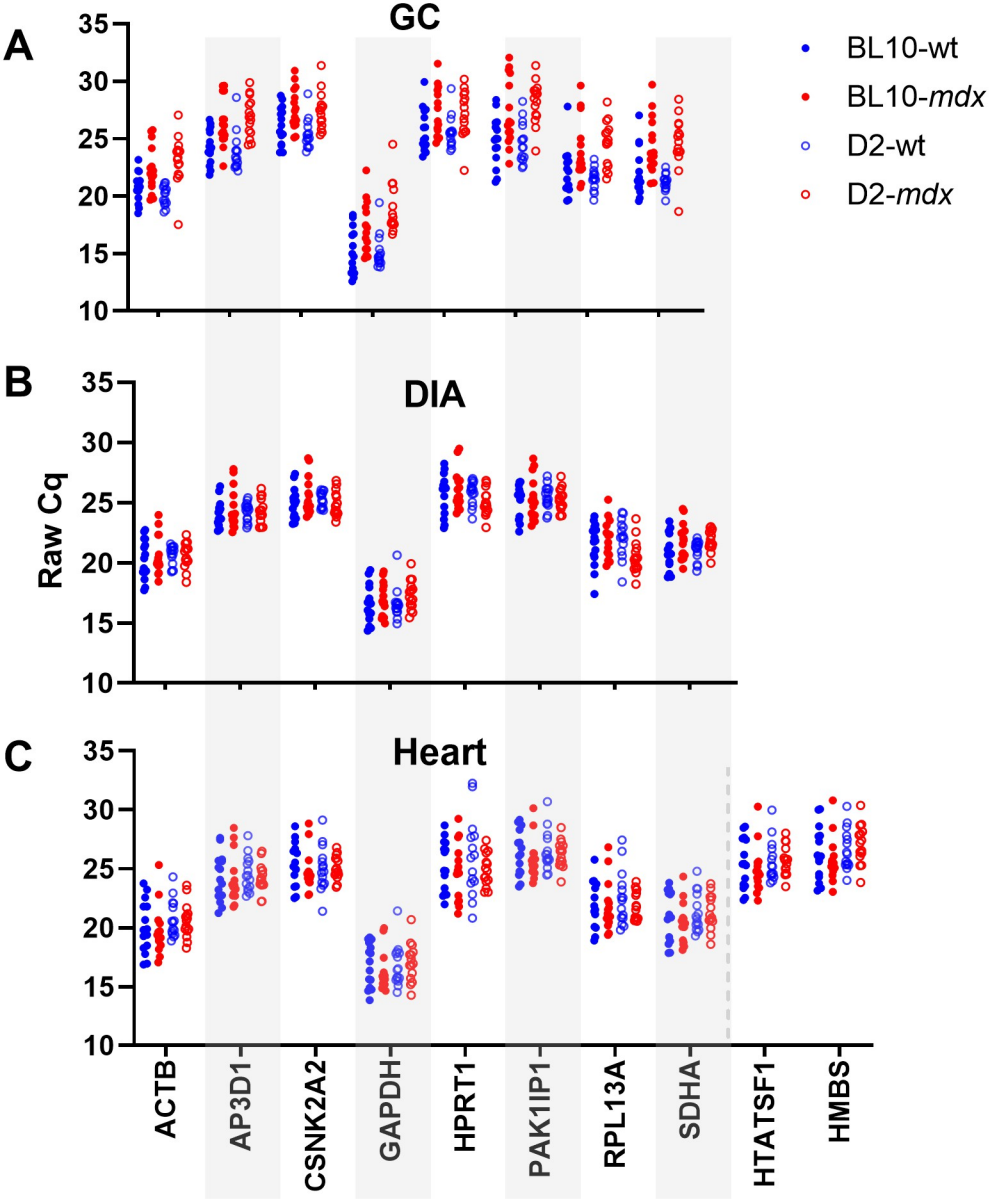
Raw Cq data for this study. Individual Cq values for each sample, for each gene (as indicated) are shown for (A) GC, (B) DIA, and (C) heart. Data is presented separated by strain: Blue closed circles: BL10-wt; Red closed circles: BL10-*mdx*; Blue open circles: D2-wt; Red open circles: D2-*mdx*. Note that for heart specifically (C) an additional two genes (*HTATSF1* and *HMBS*) were assessed.

Raw Cq data from the 180 samples within this dataset were largely consistent with expected target abundance (Fig 1), with the lowest Cq values found in the most abundant genes (*GAPDH*, *ACTB*). Overall, the genes used to generate this dataset spanned a Cq range of ~14 to 30 (equivalent to approximately 5 orders of magnitude between highest expression and lowest). For the DIA and heart (Fig 1B and 1C), data within each candidate gene were comparable between strains and between healthy and dystrophic samples, however interestingly, expression within the GC (Fig 1A) appeared to be modestly but consistently lower within dystrophic samples.

### 3.2 GeNorm

**Fig 2:**
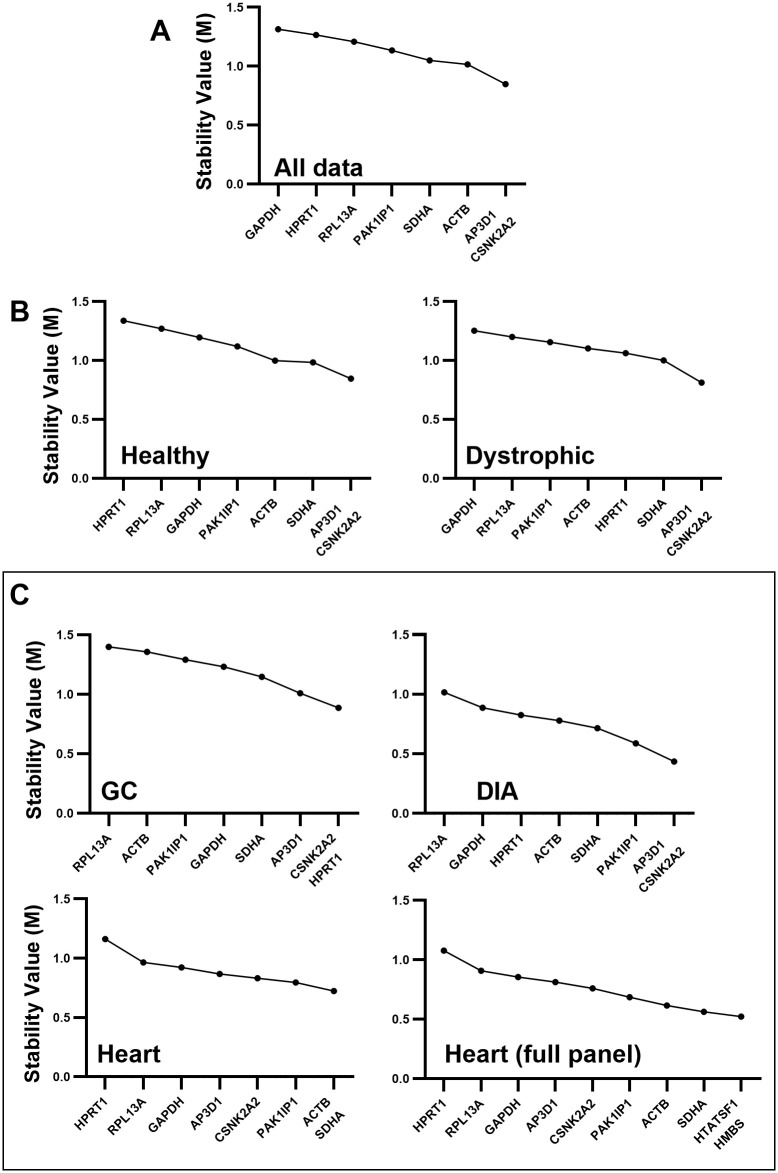
GeNorm rankings. GeNorm pairwise stability value (M) rankings for the entire dataset (A), for healthy and dystrophic subsets (B), and for individual muscle-specific subsets (C), including/excluding additional heart-specific genes. Genes are ranked from least stable (high M, lefthand side) to the most stable (low M, righthand side). As a strictly pairwise method, geNorm generates a highest scoring ‘best pair’: these two genes are considered to be of equal rank.

As shown in Fig 2A, the analysis of the entire dataset by GeNorm software identified *CSNK2A2* and *AP3D1* as the best pair (and indeed the only genes with a M value below 1.0), while *GAPDH*, *HRPT1* and *RPL13A* were the least stable (highest M). Overall stability of the dataset appeared low by this assessment approach. Typically, genes with M values <0.5 are considered suitable references, with M thresholds of <1.0 reserved only for more innately variable datasets (like primary tumour samples), however given the range of samples used here (healthy, dystrophic, three distinct muscle types, different ages and different mouse strains) such variability might be expected. Supporting this, assessment of healthy or dystrophic samples individually (Fig 2B) reduced variation (lower M values), without substantially altering ranking: *AP3D1* and *CSNK2A2* remained the best pair in both analyses. Greater decreases in overall M values were observed when samples were analysed in muscle-specific subsets (Fig 2C): while stability for the GC was comparable to the whole dataset (commensurate with the greater variation in expression within the GC shown in Fig 1A), M values were markedly lower in the heart and DIA. Muscle-specific analysis also affected gene ranking. Similar to that obtained from the entire dataset, *CSNK2A2* and *AP3D1* resulted the best pair in DIA, whereas in the GC, *CSNK2A2* was paired with *HPRT1* (one of the least stable genes in the entire dataset, and indeed in most other subsets). *ACTB* was conversely ranked as one of the least stable genes in this muscle. In the heart, *ACTB* formed part of the best pair (along with *SDHA*), however reassessment of this data with inclusion of the heart-specific candidate references, *HMBS* and *HTATSF1*, instead placed these two genes as the best pair (with yet lower M values), supporting their potential to be strong references in heart tissue.

Assessment of strain-specific subsets (S1A Fig) again ranked *CNSK2A2* and *AP3D1* as the best pair in all but BL10-*mdx*, where *ACTB* was paired with *CSNK2A2*. Assessment of age-specific subsets (S1B Fig) revealed higher M values (indicating that age might be a major factor in dataset variation for geNorm): *HPRT1* was a strong candidate at 4 weeks but scored poorly at all other ages, and indeed from 12 weeks onward rankings were largely comparable, with *CNSK2A2* and *AP3D1* consistently ranked as the best pair.

GeNorm also calculates changes in pairwise variation as number of references used increases: use of the best pair might be sufficient, but addition of further high scoring candidates can in some circumstances markedly reduce variability (and thus improve accuracy of normalisation). Typically, a variation <0.2 is considered acceptable, yet for this dataset (as perhaps suggested by the high M values, above) this was rarely achieved with the best pair alone, with use of three or more references being required to reach this threshold in most cases (particularly within the GC—S1 Table).

### 3.3 Bestkeeper

**Fig 3:**
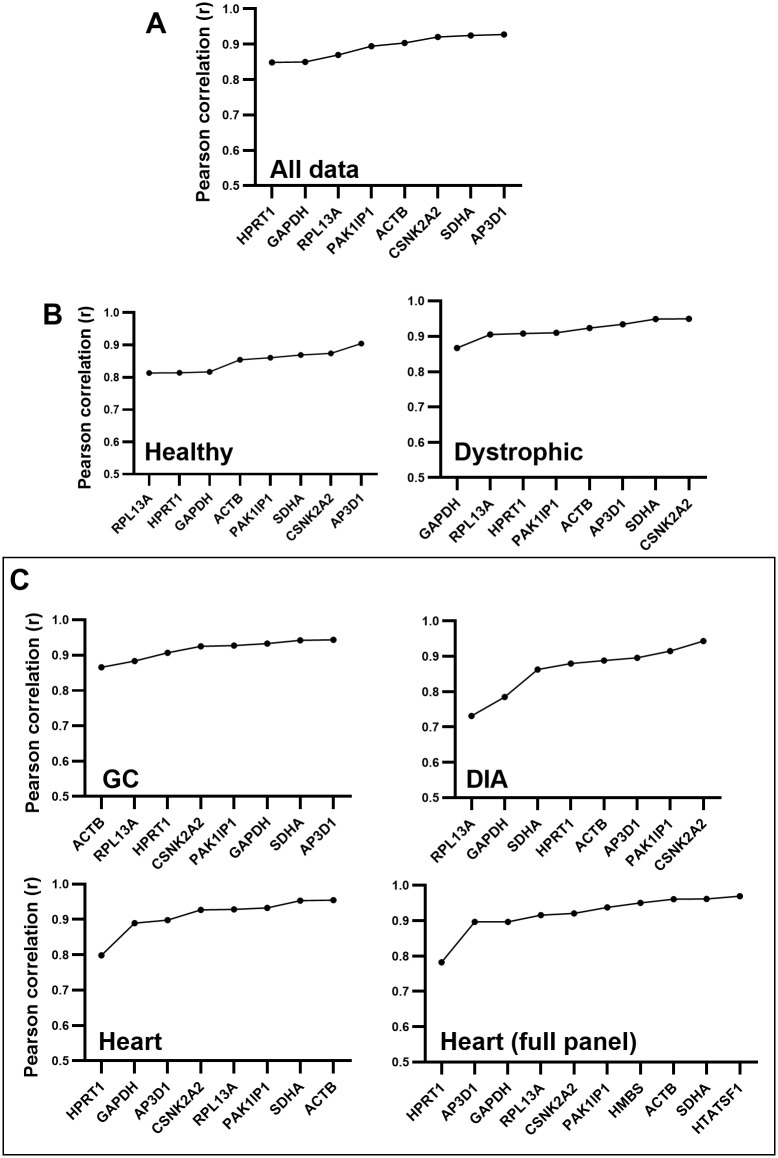
Bestkeeper rankings. Bestkeeper rankings for the entire dataset (A), for healthy and dystrophic subsets (B), and for individual muscle-specific subsets (C), including/excluding additional heart-specific genes. Genes are ranked by their coefficients of correlation (r) with the bestkeeper: low r values (lefthand side) indicate poor correlation and thus poor references, while high r values (righthand side) indicate more stable references.

Assessment of the entire dataset revealed that *AP3D1*, *SDHA* and *CSNK2A2* were the highest scoring, while *HPRT1*, *RPL13A* and *GAPDH* were ranked last (Fig 3A). Notably, however (and in contrast to geNorm), all genes were highly correlated with the bestkeeper, with even the lowest ranking candidates exhibiting correlation coefficients >0.85. In essence, any of the candidate genes would represent a suitable reference by this analysis. Analysis of healthy and dystrophic subsets retained these high correlation coefficients (Fig 3B), and similarly consistently ranked *AP3D1*, *SDHA* and *CSNK2A2* highly (albeit not identically). Gene rankings differed when muscles were assessed individually (as with geNorm): *GAPDH* and *RPL13A* exhibited markedly lower correlations than other candidate genes in the DIA, and *HPRT1* scored similarly poorly in the heart (Fig 3C), though as with analysis of the entire dataset, correlation coefficients were sufficiently high that in many cases the precise ordering of genes was otherwise largely academic. For the heart specifically, inclusion of *HTATSF1* and *HMBS* showed that both these genes did indeed score highly, with coefficients >0.94.

Assessment of data subsets by mouse strain (S2A Fig) also retained these high correlations: in the BL10-*mdx* dataset in particular, correlation coefficients of the lowest and highest scoring genes were 0.92 and 0.97, respectively.

Overall high correlation values aside, *AP3D1*, *CSNK2A2*, *ACTB* and *SDHA* were consistently scored highly by bestkeeper, essentially regardless of sample subset.

### 3.4 DeltaCt

**Fig 4:**
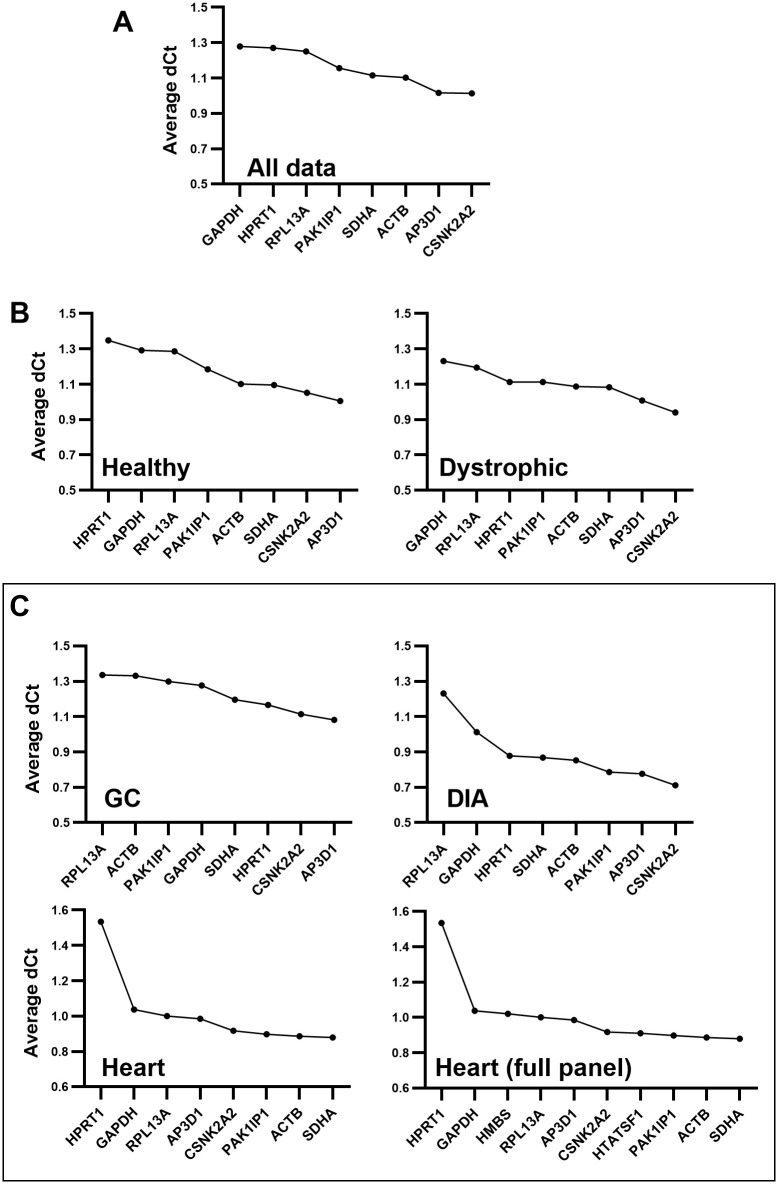
DeltaCt rankings. DeltaCt rankings for the entire dataset (A), for healthy and dystrophic subsets (B), and for individual muscle-specific subsets (C), including/excluding additional heart-specific genes. Genes are ranked by deltaCt score: the average standard deviation of dCt values (difference in expression between genes for each sample). High deltaCt scores (lefthand side) indicate more variable genes (and thus poor references), while low deltaCt scores (righthand side) indicate more stable references.

Assessment of our entire dataset (Fig 4A) suggested (in agreement with bestkeeper) that overall variation was modest: *CSNK2A2* and *AP3D1* had again the highest ranking (low deltaCt score: the mean standard deviation in dCt values), while *GAPDH* and *HPRT1* had the lowest (high deltaCt), but differences were slight, and deltaCt scores were low for all candidate genes. Assessment of healthy or dystrophic subsets alone did not significantly alter these findings (Fig 4B): again, *CSNK2A2* and *AP3D1* were high scoring, but overall deltaCt scores remained low. When subsampled by muscle type (Fig 4C), *CSNK2A2* and *AP3D1* remained high scoring (with yet further decreases in deltaCt for most genes in DIA). In the heart, conversely, *ACTB* and *SDHA* were the strongest candidates, and indeed this remained the case even with inclusion of the additional heart-specific references. Assessment by strain alone (S3 Fig) produced data essentially in agreement with assessment of the entire dataset.

### 3.5 Normfinder

**Fig 5:**
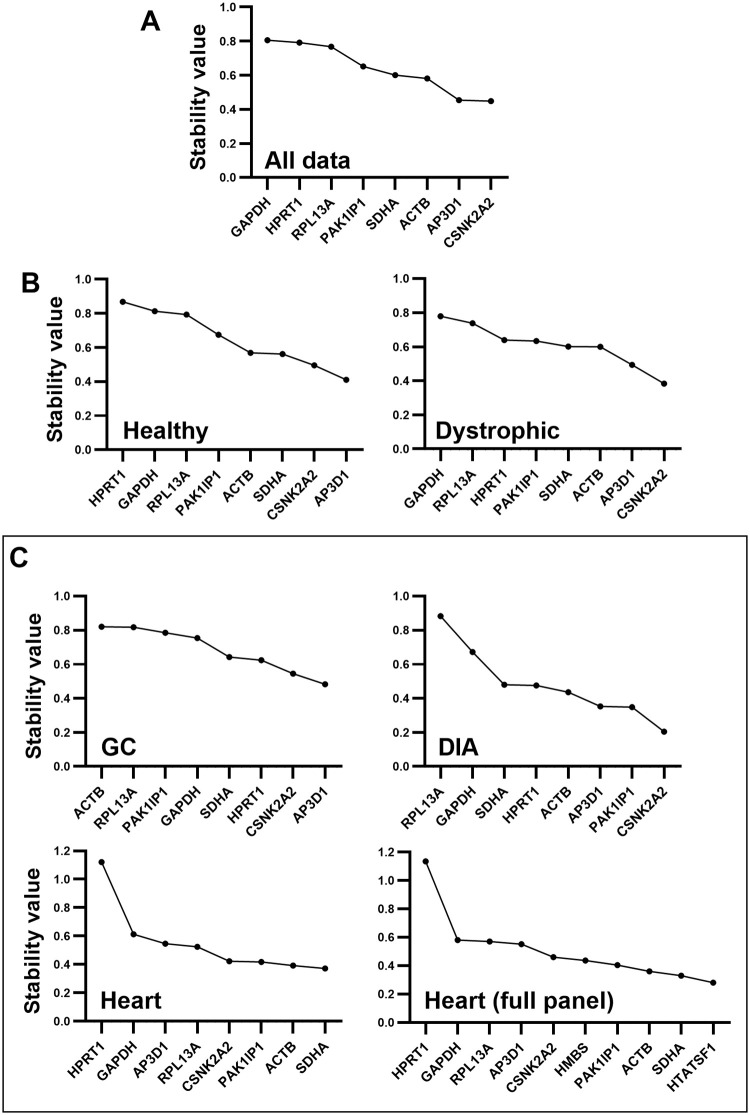
Normfinder rankings (ungrouped). Ungrouped normfinder rankings for the entire dataset (A), for healthy and dystrophic subsets (B), and for individual muscle-specific subsets (C), including/excluding additional heart-specific genes. Genes are ranked by overall expression stability: high stability values (lefthand side) indicate poor references, while low stability values (righthand side) indicate more stable references.

Assessment of the entire dataset by ungrouped analysis (Fig 5A) again identified *CSNK2A2* and *AP3D1* as the most stable, followed by *ACTB* and *SHDA*, while *GAPDH*, *HRPT1* and *RPL13A* scored particularly poorly. A similar pattern was observed with healthy or dystrophic subsets (Fig 5B): *CSNK2A2* and *AP3D1* scored highest, with *GAPDH*, *HPRT1* and *RPL13A* consistently ranked last. Muscle-specific subsets (Fig 5C) refined these findings: both *AP3D1* and *CSNK2A2* remained high scoring within the skeletal muscles, but rankings of other genes varied with muscle type. *ACTB* was relegated to the lowest ranking in the GC, while *HPRT1* was ranked comparatively highly. As shown by other methods (above) stability within the DIA was also high overall: *PAK1IP1* ranked highly with *CSNK2A2* but, in general, only *GAPDH* and *RPL13A* were notably low scoring. In agreement with other analyses, heart-specific ranking was different: restricted to the core 8 genes of the reference panel, *CSNK2A2* and *AP3D1* were of only modest stability, with *ACTB* and *SDHA* being the most stable. Inclusion of the two heart-specific reference genes ranked *HTATSF1* highest (ahead even of *SDHA*), but here *HMBS* was comparatively poorly scored. Assessment of strain-specific datasets (S4 Fig) suggested that only modest differences between mouse lines: *AP3D1* and *CSKN2A2* were ranked highest universally, while *RPL13A*, *GAPDH* and *HPRT1* predominantly (but not universally) scored poorly.

**Fig 6:**
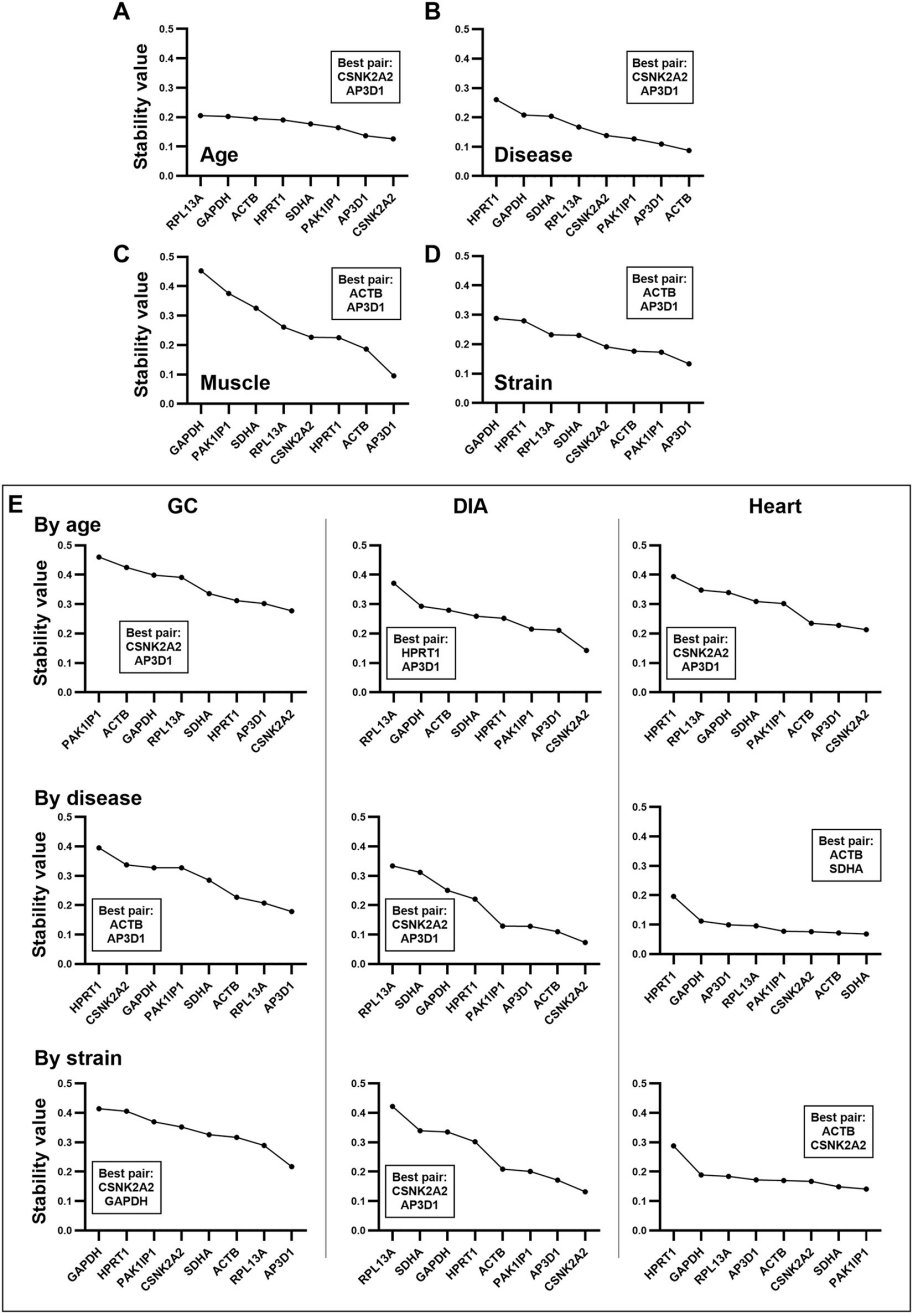
Normfinder rankings (grouped). Normfinder can assess expression stability between and within user-specified groups. Grouped normfinder rankings are shown for the entire dataset grouped by age (A), disease (B), muscle type (C) or strain (D), and for (E) muscle specific datasets (as indicated) grouped by age (top row), disease (middle row), or strain (bottom row). Genes are ranked by overall expression stability: high stability values (lefthand side) indicate poor references, while low stability values (righthand side) indicate more stable references. Grouped analysis also provides a ‘best pair’ -two genes that combined provide greater stability than any individual gene: these are shown on each plot (inset boxes).

To refine these findings, we then conducted grouped analysis, using the entire dataset (grouped by age, disease, muscle type or strain) and muscle-specific datasets (grouped age, disease or strain). Analysis of the entire sample set grouped by age (Fig 6A) yet again assigned the highest rankings to *AP3D1* and *CSNK2A2*, and the lowest to *RPL13A* and *GAPDH*, however notably all genes were comparatively stable under this grouping (stability values < 0.25), suggesting that none of the candidate references is substantially affected by age. Grouped analysis also provides a “best pair”: a pair of genes that provide a greater stability value, combined, than any single gene alone. While this best pair need not be comprised of the two highest ranking genes, *CSNK2A2* and *AP3D1* formed the best pair for the entire dataset grouped by age. When grouped by healthy/dystrophic (Fig 6B), *ACTB* was the most stable individual gene, but *CSNK2A2* and *AP3D1* again formed the best pair. Grouping by muscle type lowered overall dataset stability (indicating that muscle type represents a greater source of variation than age or dystrophy -Fig 6C), and here *AP3D1* was the strongest candidate, but joined now by *ACTB*, rather than *CSNK2A2* (indeed *ACTB* and *AP3D1* formed the best pair for this grouping). *GAPDH* again scored poorly, as did *PAK1IP1* but also *SDHA*. Finally, grouping by strain again showed the *AP3D1* was the strongest candidate, and *AP3D1*/*ACTB* were the best pair (*GAPDH* was ranked last) (Fig 6D).

Muscle-specific grouped analysis by age (Fig 6E, top row) revealed that *CSNK2A2* and *AP3D1* were the highest scoring regardless of muscle type (though were not always also the best pair). Individual rankings of other candidates -including *ACTB*- were more variable, however. Grouping by healthy/dystrophic (Fig 6E, middle row) indicated considerable heterogeneity between muscles: in the GC, *RPL13A* was high scoring while *CSNK2A2* scored poorly, in the DIA *CNSK2A2* and *AP3D1* were again strong candidates, while in the heart essentially all genes were highly stable except for *HPRT1*. These findings were largely mirrored when data were grouped by strain (Fig 6E, bottom row), suggesting that healthy/dystrophic distinctions contribute to variation more than individual strain differences. One interesting finding of this latter analysis was that within the GC specifically, *GAPDH* was both the least stable gene and also one of the best pair (along with *CSNK2A2*), implying that expression of these two genes varies in an opposed (but highly consistent) fashion such that their average behaviour is more stable even than the most stable single gene. Heart samples reassessed with the inclusion of *HMBS* and *HTATSF1* again scored *HTATSF1* highly regardless of group designation (S5A Fig) and assessment of all healthy (or dystrophic) samples grouped by age, muscle or strain was largely in agreement with the combined (healthy plus dystrophic) dataset, with *AP3D1* and *CSNK2A2* typically scoring highly under all groupings (S5B Fig).

### 3.6 Aggregate rankings

**Fig 7:**
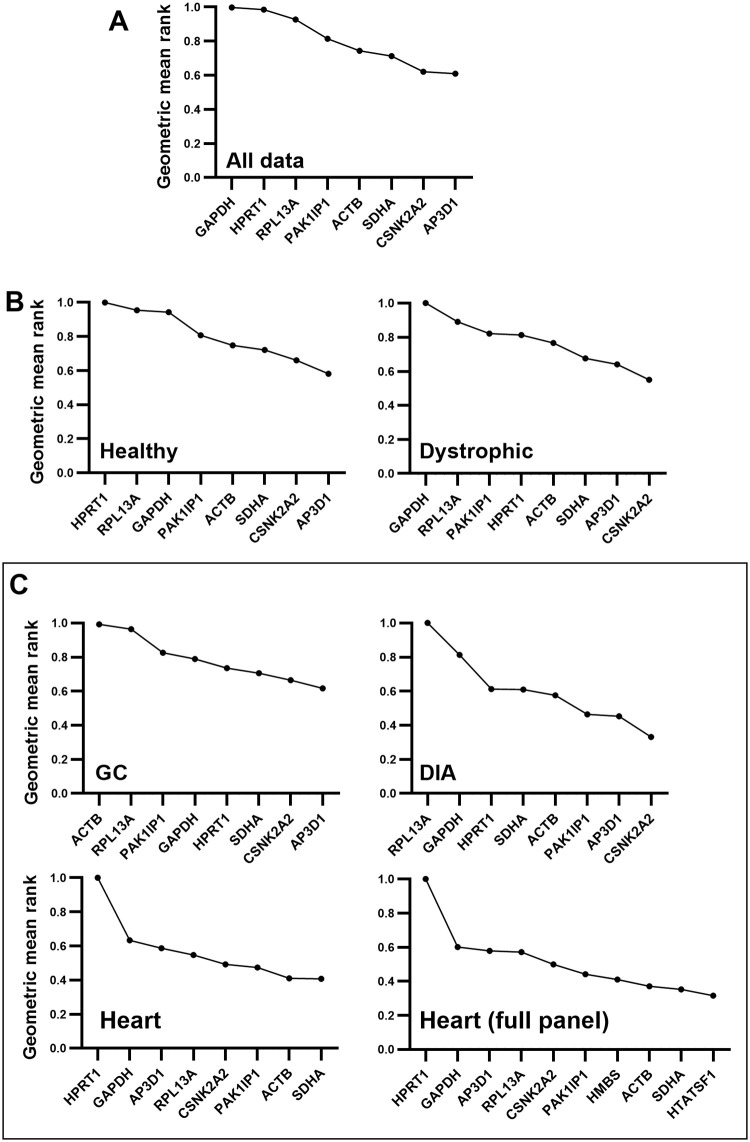
Aggregate rankings. Geometric mean values incorporating individual rankings of all four assessment methods, for the entire dataset (A), for healthy and dystrophic subsets (B), and for individual muscle-specific subsets (C), including/excluding additional heart-specific genes. All assessment method values are converted to a 0–1 linear scale (see Methods) where high values indicate low stability (lefthand side) and low values indicate high stability (righthand side).

Given their strong performance within each assessment approach, *CNSK2A2* and *AP3D1* were unsurprisingly ranked highest (Fig 7A), with *SDHA* and *ACTB* also scoring highly (Fig 7B), suggesting that these four genes are suitable references for all muscle samples irrespective to genotype. Consensus scores for specific muscles were in less agreement (Fig 7C), supporting that muscle type is a major source of between-sample variation within this dataset. *CSNK2A2* and *AP3D1* remained strong in both the GC and DIA, but *ACTB* was the lowest ranking gene in the former muscle, while comparatively strong in the latter. Indeed, many of the candidate genes exhibited relatively stable expression in DIA. A different consensus ranking was found in the heart: *ACTB* and *SDHA* were the genes with the highest score (in line with their high performance in each individual approach), while here *CNSK2A2* and *AP3D1* scored more poorly. Inclusion of the two heart-specific references in the consensus ranking placed *HTATSF1* emphatically ahead of even *SDHA* and *ACTB*, while *HMBS* placed behind these two genes.

*CSNK2A2* and *AP3D1* appear to be strong reference genes, with *ACTB* and *SDHA* also potentially representing viable candidates. GeNorm analysis indicated substantial decreases in pairwise variation using three, rather than two reference genes. Evaluation of raw Cq data (Fig 1) indicates that expression of *CSNK2A2* and *AP3D1* is modest (mean Cq values of 25–26): while this demonstrably does not affect their scoring as strong references, quantification of lower abundance transcripts is inherently more vulnerable to noise. Expression of *ACTB* is conversely more robust, expressed at levels ~10-20-fold higher, and thus inclusion of this gene in a reference panel might be expected to buffer against stochastic variation (*SDHA* is also abundant, but previous data has suggested prominent muscle-specific expression patterns for *SDHA*, but not *ACTB*, in mouse [25]. We note that while *ACTB* was poorly ranked in GC specifically, it scored highly in this tissue when grouped by disease or strain (Fig 6), implying it is largely stable with respect to these factors.

**Fig 8:**
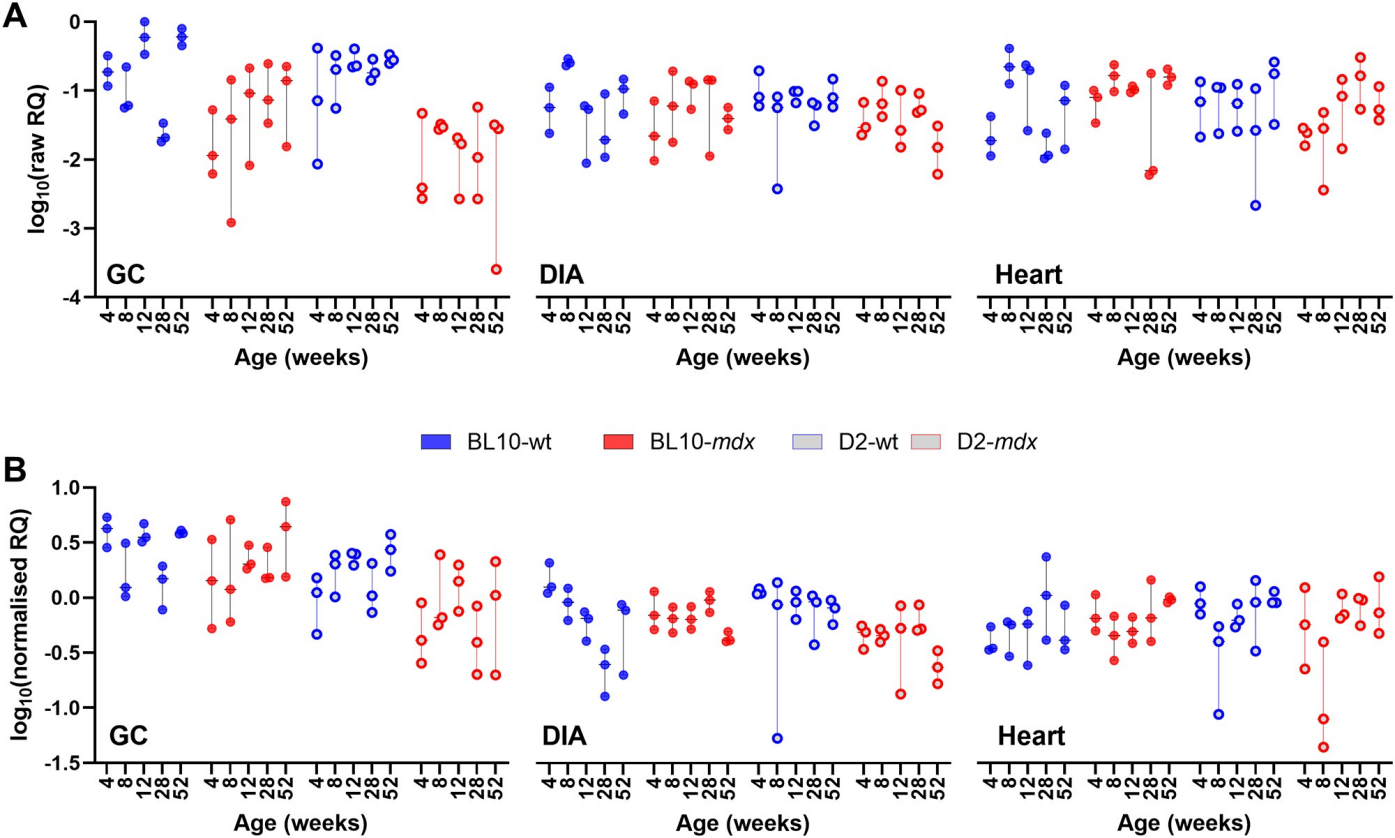
Normalisation of *GAPDH*. Gene expression of *GAPDH* across the dataset without normalisation (A) and following normalisation to the geometric mean of *AP3D1*, *CSNK2A2* and *ACTB* (B). Data is shown as log10 of relative quantity (RQ) values (non-normalised or normalised, respectively). Mean per-group coefficient of variation is reduced following normalisation (0.68 raw, 0.44 normalised) and normalised data suggests modest but consistent muscle-specific differences in expression.

**Fig 9:**
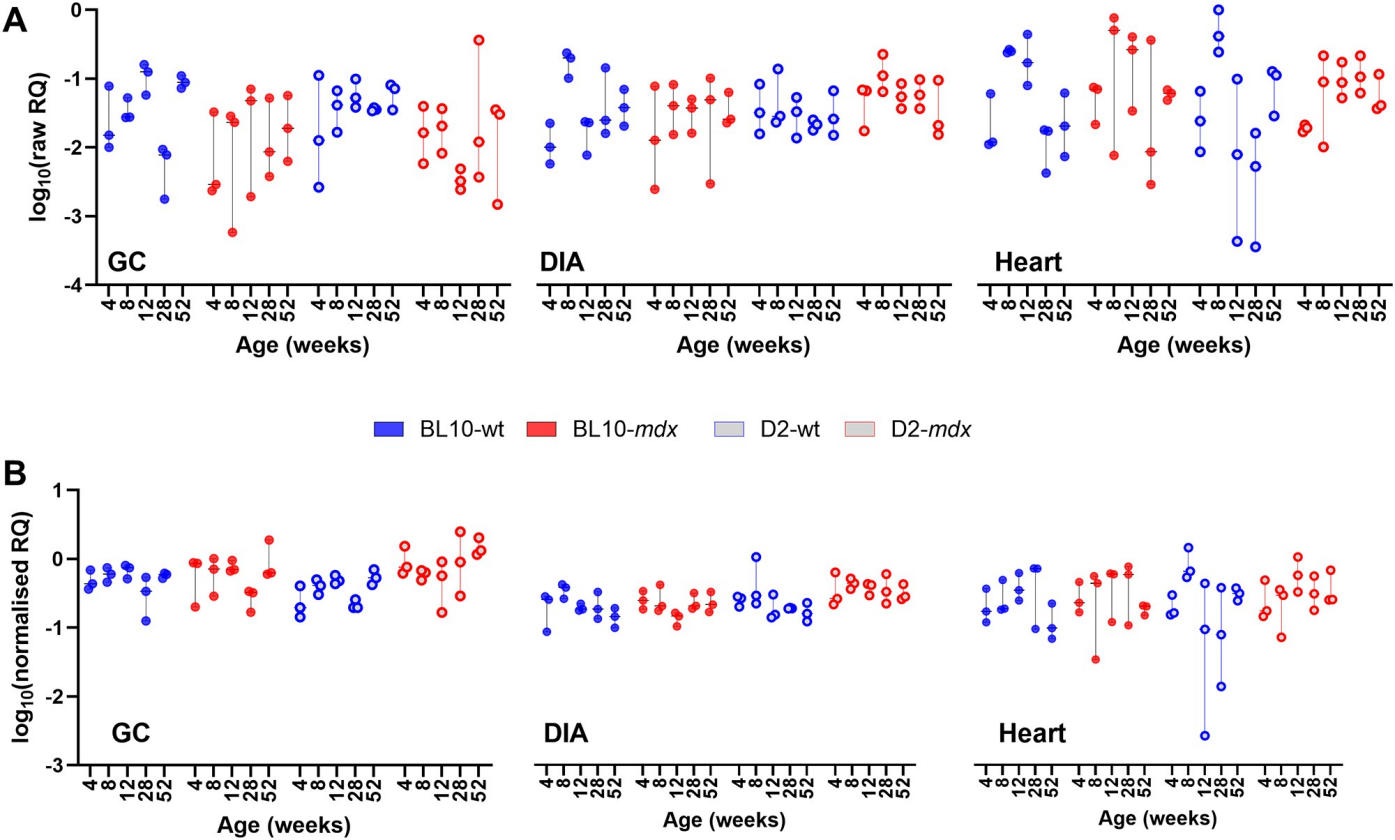
Normalisation of *HPRT1*. Gene expression of *HPRT1* across the dataset without normalisation (A) and following normalisation to the geometric mean of *AP3D1*, *CSNK2A2* and *ACTB* (B). Data is shown as log10 of relative quantity (RQ) values (non-normalised or normalised, respectively). Mean per-group coefficient of variation is reduced following normalisation (0.74 raw, 0.49 normalised) and normalised data suggests modest but consistent muscle-specific differences in expression, and high innate variability within heart.

**Fig 10:**
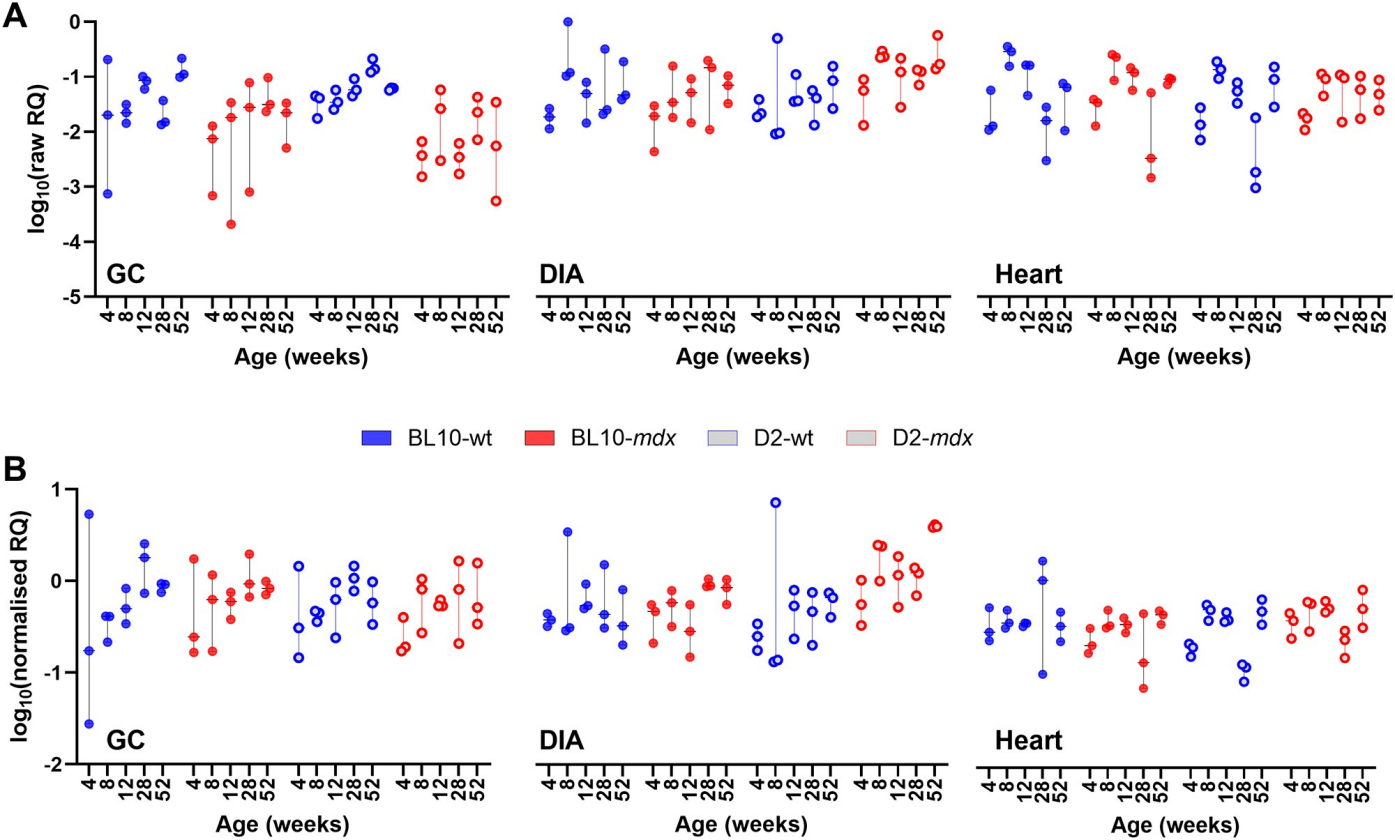
Normalisation of *RPL13A*. Gene expression of *RPL13A* across the dataset without normalisation (A) and following normalisation to the geometric mean of *AP3D1*, *CSNK2A2* and *ACTB* (B). Data is shown as log10 of relative quantity (RQ) values (non-normalised or normalised, respectively). Mean per-group coefficient of variation is reduced following normalisation (0.69 raw, 0.48 normalised) and normalised data suggests broadly comparable muscle-specific expression, but a modest yet consistent increase in expression with age.

To evaluate the utility of *CSNK2A2*, *AP3D1* and *ACTB* as a candidate reference panel, we employed a strategy we have used historically: using our high scoring candidate genes to normalise expression of those that scored most poorly. In this dataset, *GAPDH* and *HPRT1* were near-uniformly ranked last, with *RPL13A* also typically ranking poorly in most assessments. A comparison of raw *GAPDH* data expression data (Fig 8A) with that normalised to our 3-gene panel (Fig 8B) suggests that this gene shows muscle-specific expression, being more abundant in the GC muscle than the heart or DIA. Expression was also consistent with modest but consistent dystrophy-associated reductions in D2-wt/D2-*mdx* samples. Effective normalisation should reduce overall variation between replicate samples (same age, muscle and strain), and the mean per-group coefficient of variation (CoV) of data was indeed markedly reduced by normalisation in this manner (from 0.68 to 0.44). Similarly, normalisation of *HPRT1* to *CSNK2A*/*AP3D1*/*ACTB* resulted in a stabilisation of replicate group variation (CoV 0.74 to 0.49) and suggested that expression of *HPRT1* is not markedly different across strains and genotypes, but nevertheless (like *GAPDH*) varies in a muscle-specific fashion (Fig 9): being more abundant in GC, and more variable in heart. Finally, normalisation of *RPL13A* using our 3-gene panel reduced CoV (0.69 to 0.48) and revealed this gene to be moderately associated with age, with normalised data consistently increasing in expression from 4 weeks to 52 weeks in both GC and DIA (and further upregulated in dystrophic D2-*mdx* DIA specifically -Fig 10). As a final test, we also normalised *ACTB* to the geometric mean of *CSNK2A2* and *AP3D1*. Again, this reduced replicate CoV (0.65 to 0.40), and while variation remained considerable in the GC (as expected), no clear association with age, disease or muscle type was observed, supporting use of ACTB as a reference (S6 Fig).

## 4. Discussion

This study aimed to identify the most suitable reference genes for gene expression analyses in the D2-*mdx* mouse, a newly introduced, yet still under-characterized murine model for DMD, throughout its natural history. Identifying appropriate reference genes is crucial for obtaining accurate results in gene expression analyses and gaining a better understanding of the functioning of this newly developed murine model.

Taken together, the data presented here makes a convincing case for *CSNK2A2* and *AP3D1* being very strong reference genes for qPCR normalisation in mouse skeletal muscle: these two genes were near-unanimously ranked highest regardless of assessment method used. This strong performance is perhaps not unexpected: we have shown these genes are suitable references in mouse muscle previously [25]and also in murine myogenic cell lines [29], but the work here refines these earlier findings by assessing additional time points [covering a significant fraction of animal lifespan], and extending investigations to a new dystrophic mouse model with greater disease severity. This earlier work similarly identified *ACTB* as a strong candidate: again our work here further supports this. It should be noted that two previous studies [29, 30] have suggested that ACTB is a poor reference for normalizing expression during regeneration after induced muscle injury: this apparent discrepancy likely reflects differences between acute regeneration of otherwise healthy muscle, and the chronic asynchronous degeneration-regeneration affecting dystrophic muscles in the murine model [31, 32].

The high scoring of *SDHA* is less aligned with prior findings, where this gene was found to vary substantially between muscle groups in the mouse (though we note this gene is a strong candidate in healthy and dystrophic canine muscle [26]). Fewer muscle groups were examined in this study, however, and *SDHA* further performed particularly strongly within the heart, which might have bolstered the overall ranking of this gene within the dataset.

Similarly, it is difficult to argue that *GAPDH* and *HPRT1* are, here, emphatically rejected as suitable reference genes: these genes were near universally ranked last and second last, often by a substantial margin. *RPL13A* also performed poorly, albeit more variably, suggesting this too represents a poor choice for normalisation in mouse skeletal muscle. We and others have shown that *GAPDH* is a poor reference in many contexts (including muscle) and the data here adds to this growing body of evidence. *HPRT1* and *RPL13A*, conversely, are both strong candidates for normalising gene expression in canine muscle, and previous data suggested *RPL13A* was also suitable for healthy and dystrophic mouse muscle [25]. The low scores of these latter genes are thus somewhat unexpected. Our validation strategy (using strong reference candidates to normalise weak candidates) suggests reasons for these low rankings: for *GAPDH*, expression varies substantially across different muscle groups (and in the GC and DIA, might also modestly decline with age in healthy, but not dystrophic samples -Fig 8B). *HPRT1* also exhibits distinct muscle-specific behaviour (Fig 9B), and although this gene appears comparatively stable within individual skeletal muscles, expression is highly variable in heart [notably, *HPRT1* was ranked last in heart by every single approach). Finally, *RPL13A* unexpectedly appears to exhibit consistent increases in expression with age: we note that while our previous study examined a larger collection of muscle groups, samples were collected only from WT (BL10-wt) and BL10-*mdx* mice, and from only three ages (6, 10, 24 weeks). It is likely that the greater number of samples used here, capturing expression changes from 4 weeks through to a full 52 weeks of age, allow these modest but consistent age-related changes to be revealed.

Assessment of gene expression in the heart merits further attention: here we included an additional two candidates, *HMBS* and *HTATSF1* (the latter we have shown previously to be a strong candidate in heart specifically [26]). Our data here confirms this gene as being highly appropriate for normalising expression within cardiac muscle: assessment of the dataset with these additional genes included ranked *HTATSF1* as the strongest candidate in almost all instances. Performance of *HMBS* was however less impressive: while this gene was typically one of the better scoring candidates, it rarely ranked higher than *ACTB*, *CNSK2A2* or *AP3D1*. Why *HTATSF1* should be so stably expressed within heart tissue specifically is not clear: we did not examine expression of this gene in the GC or DIA (previous data suggested it was unlikely to score highly in a wider context), but strong stability within heart specifically is not restricted to this gene: *SDHA* (as discussed above) was also ranked as highly stable in heart, but more modestly stable in other muscles. The heart is a highly specialised muscle, but also arguably a more homogenous tissue than striated muscles, where a mosaic of fibre types with differing oxidative capacities are present: this homogeneity might be reflected in the behaviour of some, but not all, genes.

Our data here suggests that for studies of the heart specifically, *HTATSF1* and *SDHA* would serve as better references than *CSNK2A2*, *AP3D1* and *ACTB*, but we note that these latter three genes are still strong candidates in heart tissue. For studies addressing heart alongside skeletal muscles, therefore, where a common set of references would be more advantageous, *CSNK2A2*, *AP3D1* and *ACTB* would serve as such a universal panel.

It is also important to recognise that the genes used for this analysis were primarily selected based on prior strong performance in skeletal muscle specifically or in qPCR normalisation overall: accordingly, while our analysis shows consistent muscle- or age-specific differences in expression for some genes, the bulk of our candidate genes still scored comparatively highly overall, by most empirical assessment metrics. *GAPDH*, *HPRT1* and *RPL13A* are demonstrably not appropriate universal references for skeletal muscle analyses, but usually by a substantial margin: beyond avoidance of these three, essentially any of the remaining genes would serve as adequate references.

In fact, the coefficients of correlation reported here are markedly higher than those we have observed historically. DeltaCt values imply modest stability, and values reported by Normfinder are (like geNorm) indicative of low stability when data is assessed ungrouped, but stability increases markedly with grouped analysis, with the exception of grouping by muscle. The methods used by each approach to quantify stability are not equivalent: indeed, these critical differences enhance the power of using multiple methods, as shown here. Pertinent to this analysis, geNorm uses an iterative approach, eliminating candidate genes progressively, but ranking each by their pairwise score at time of elimination. An explanation we propose, therefore, is that the dataset we have used contains multiple strong reference gene candidates, as noted above, but also exhibits significant innate sample-to-sample variation, i.e. substantial variation in mRNA content between samples -not unexpected when comparing healthy and dystrophic muscle samples. GeNorm analysis of such a dataset will thus report comparatively high pairwise variation persistently, as this variation predominantly reflects differences in samples, not in gene expression. Bestkeeper conversely ranks by correlation with the consensus behaviour of all genes within a dataset: variation between samples is not strictly assessed, only the per-sample variation of each individual gene from the average expression within that sample. Here the same dataset would thus report very high correlations: all candidate genes are strong, regardless of innate sample-to-sample variation. The deltaCt method assesses differences between individual genes on a per-sample basis, but then also averages differences across all gene comparisons: this approach thus falls somewhat between these two extremes. Normfinder will report high variation overall reflecting the high sample-to-sample variation, but when samples are assigned to groups with potentially more consistent within-group expression, reported variation will be much lower. Use of four complementary assessment methods thus both identifies clear front running gene normalisation candidates, but also can reveal subtle facets to dataset behaviour as a whole.

## 5. Conclusions

We have conducted a broad and comprehensive examination of candidate reference gene expression stability, using multiple muscles of multiple mouse strains, both healthy and dystrophic, collected at different ages. We show that *ACTB*, *CSNK2A2* and *AP3D1* represent a strong universal reference panel for normalising qPCR data in murine skeletal muscle, regardless of age, muscle type, strain or presence of dystrophic pathology, and we further show that for studies focussing on the heart specifically, *HTATSF1* and *SDHA* represent a yet stronger combination of references. Our results provide a solid basis for further comparison of best reference genes in the mdx mouse models within the results that will be obtained with the same approach and independently in LUMC, in the frame of the common “Of Mouse and Measures” (OMAM) project, so to strengthen the qPCR data for the exploratory genes of interest and implement Standard Operating Procedures for preclinical gene expression studies in muscular dystrophy.

## Supporting information

S1 Fig Assessment of strain- and age-specific subsets with GeNorm. (TIF)

S2 Fig Assessment of data subsets by mouse strain with BestKeeper. (TIF)

S3 Fig Assessment by strain alone with DeltaCt. (TIF)

S4 Fig Assessment of strain-specific datasets with Normfinder. (TIF)

S5 Fig Assessment of all healthy (or dystrophic) samples grouped by age, muscle or strain with Normfinder. (TIF)

S6 Fig Normalization of *ACTB* to the geometric mean of *CSNK2A2* and *AP3D1*. (TIF)

S1 Table Changes in pairwise variation with GeNorm. (DOCX)

S1 Data Raw Cq data. (XLSX)
