# Supplementary figures and images for: Determination of qPCR reference genes suitable for normalizing gene expression in a novel model of Duchenne muscular dystrophy, the D2-mdx mouse
Source: PLoS One. 2024 Nov 13;19(11):e0310714. doi: 10.1371/journal.pone.0310714 (PMC11560031; doi:10.1371/journal.pone.0310714)

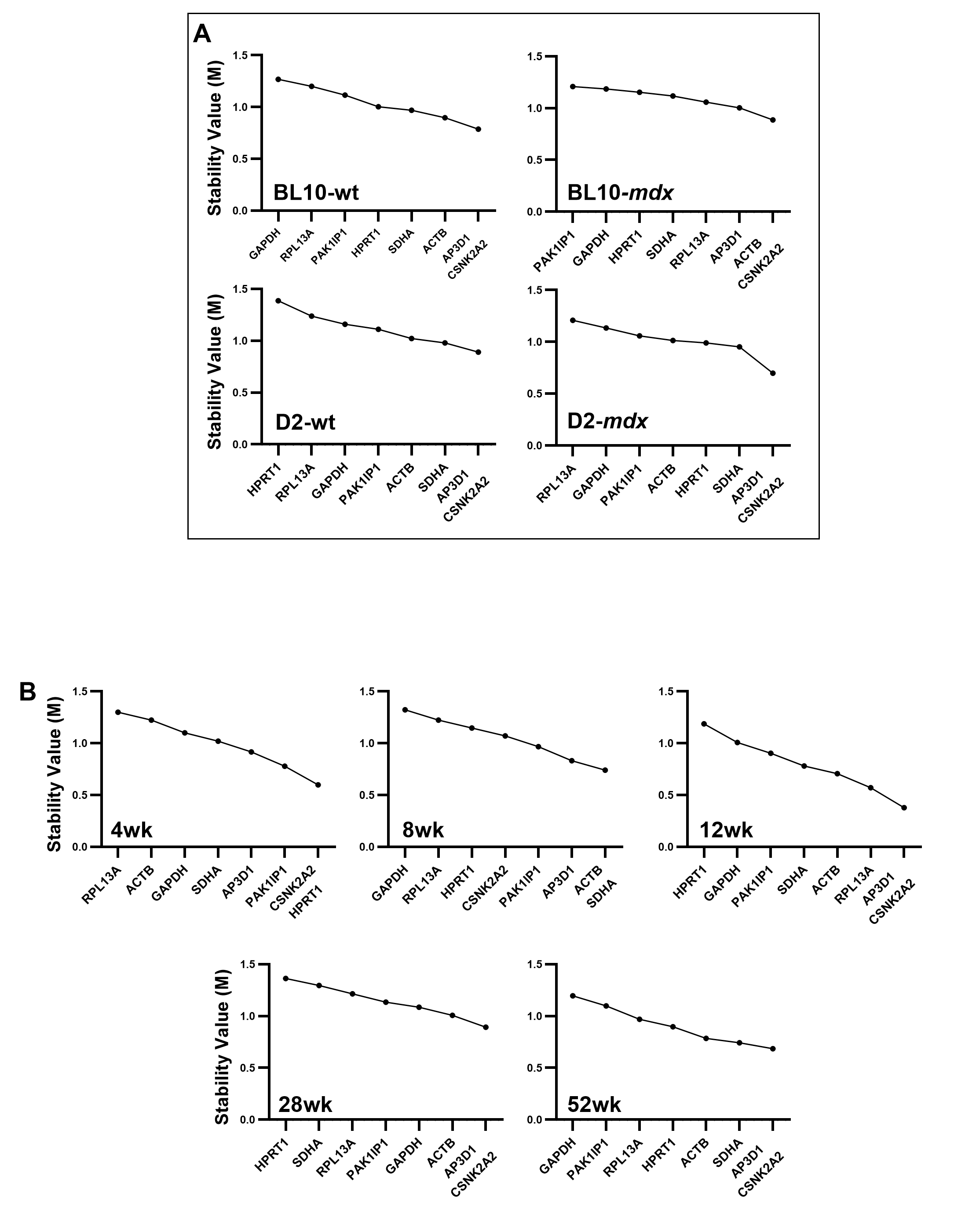

Supplement: S1 Fig — (TIF) [file pone.0310714.s001.tif]

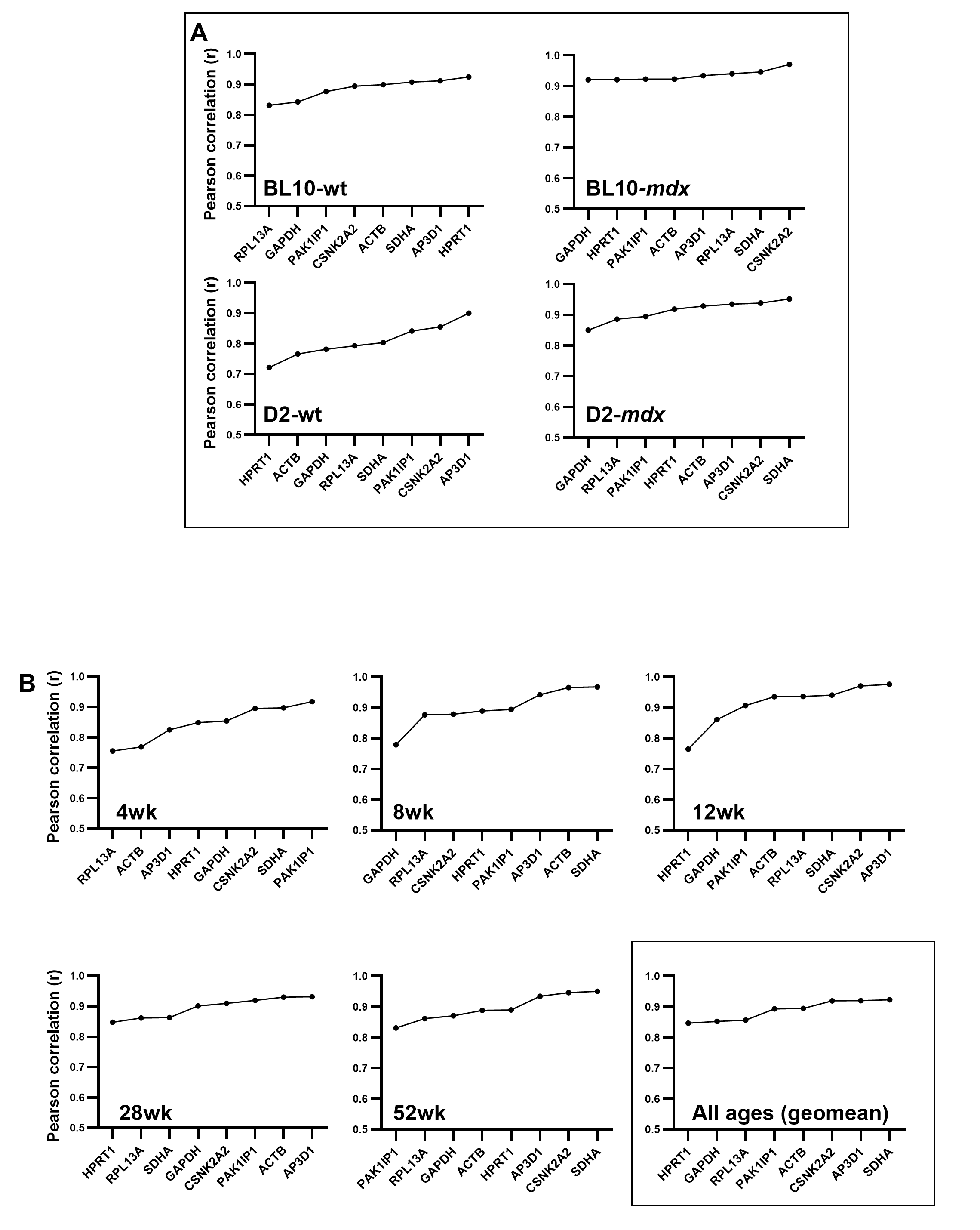

Supplement: S2 Fig — (TIF) [file pone.0310714.s002.tif]

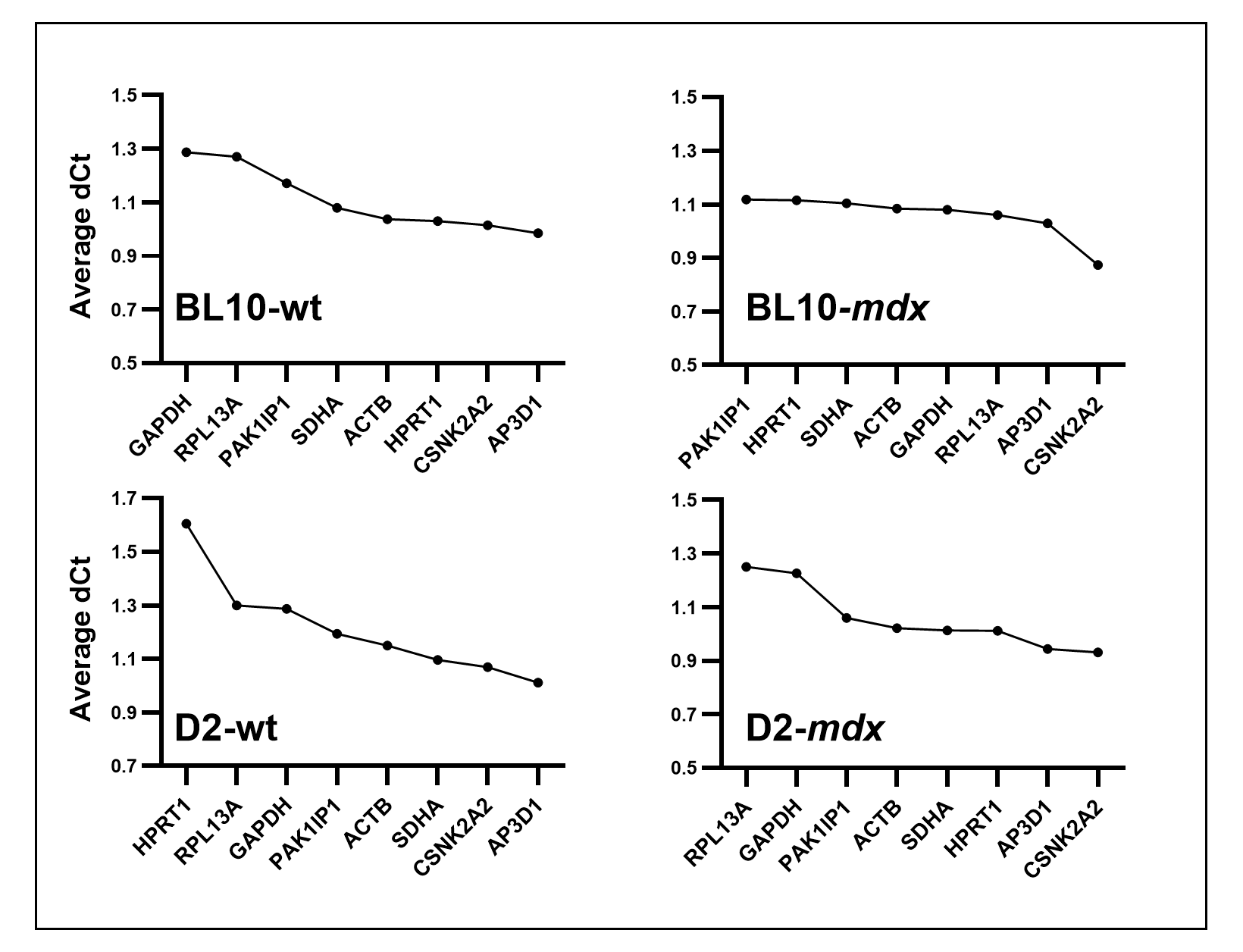

Supplement: S3 Fig — (TIF) [file pone.0310714.s003.tif]

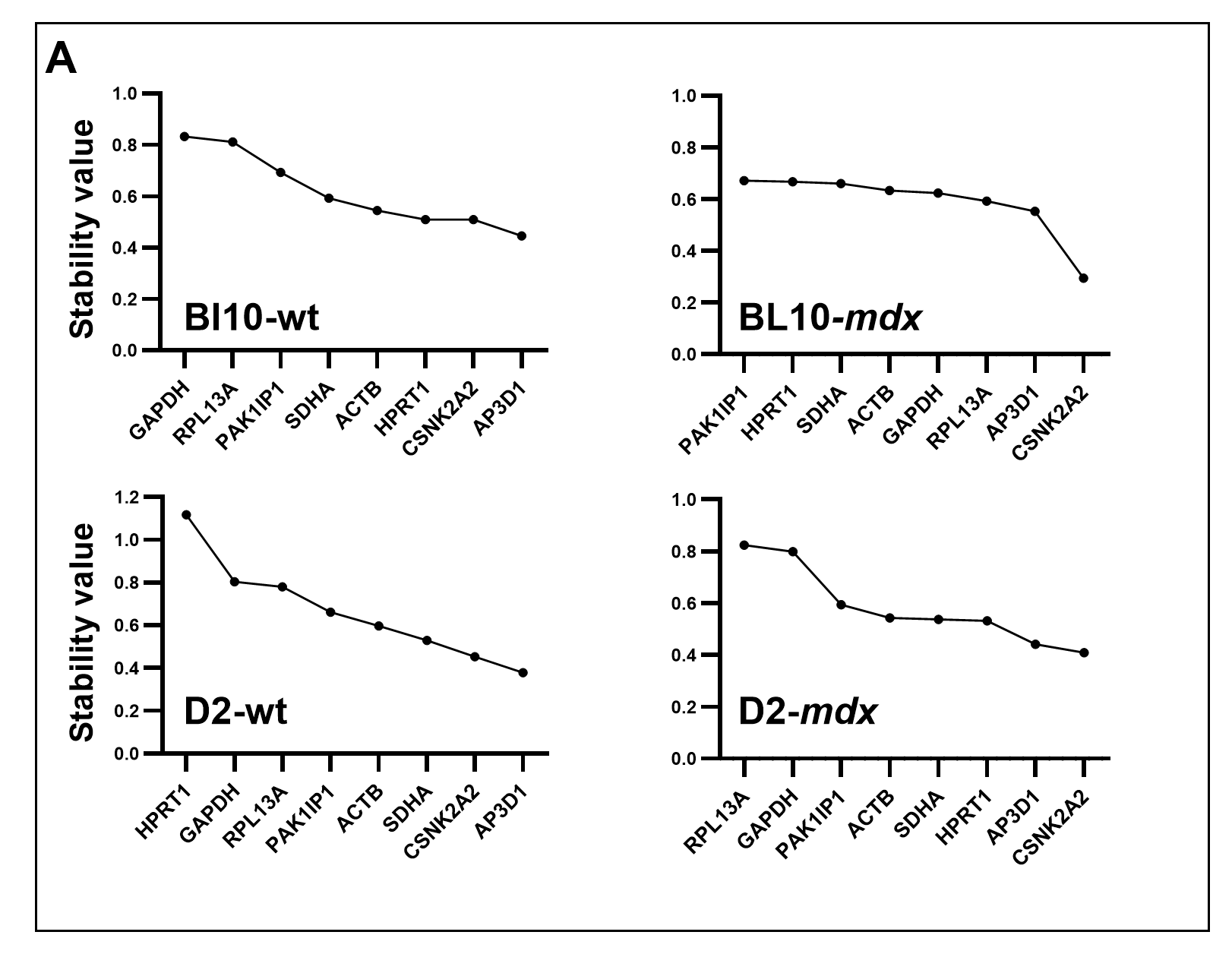

Supplement: S4 Fig — (TIF) [file pone.0310714.s004.tif]

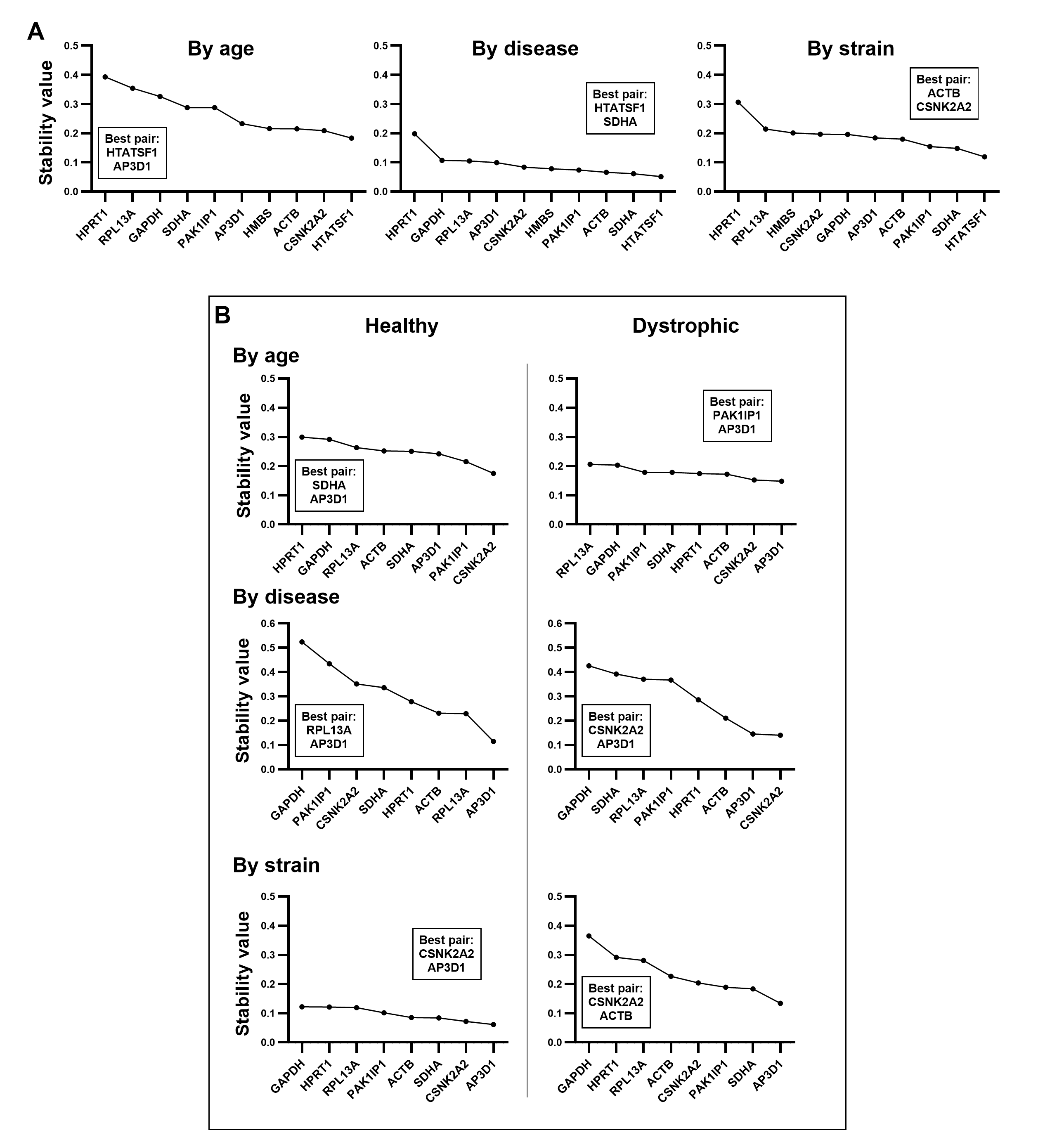

Supplement: S5 Fig — (TIF) [file pone.0310714.s005.tif]

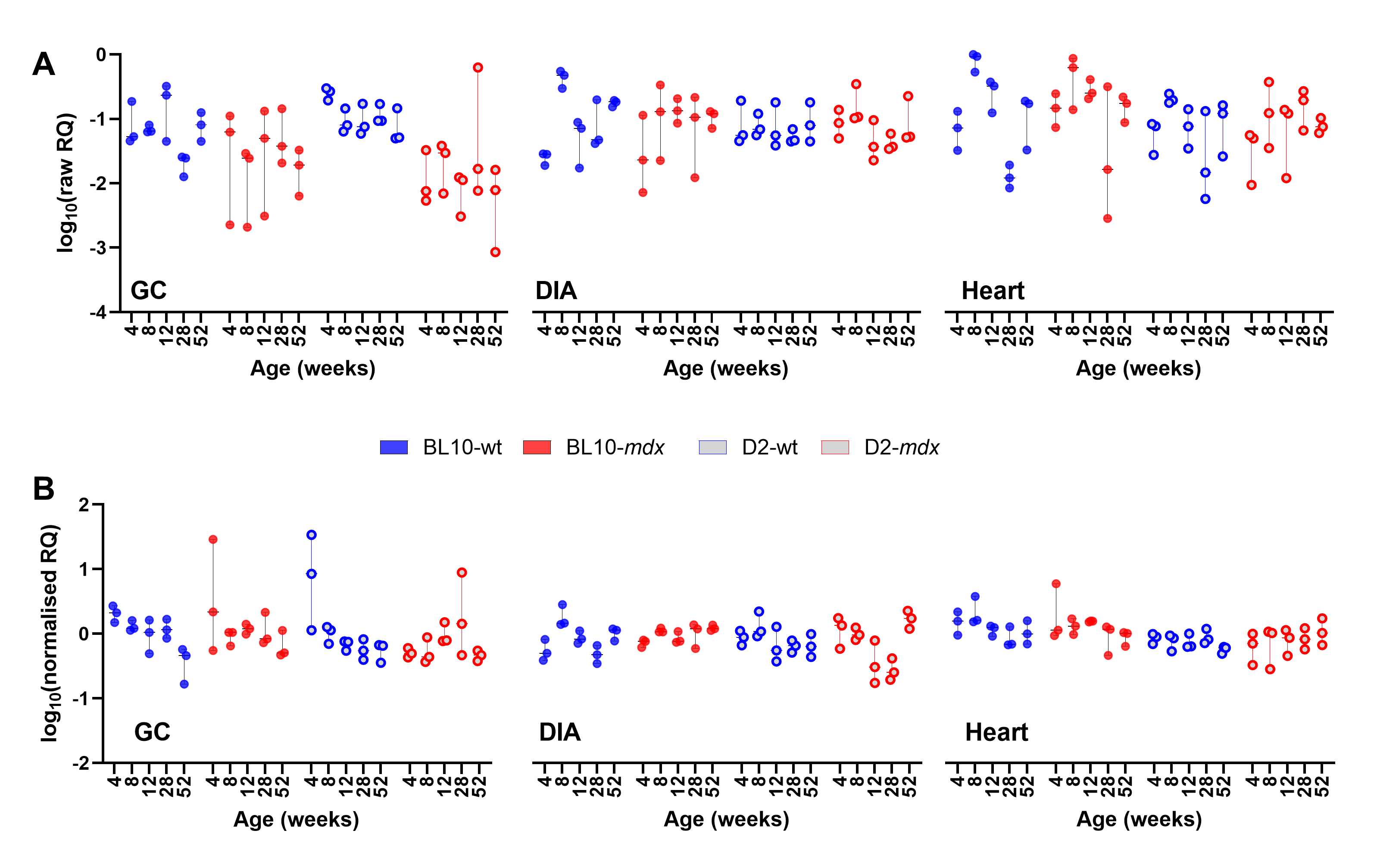

Supplement: S6 Fig — (TIF) [file pone.0310714.s006.tif]
