# Supplementary material for: Determination of qPCR reference genes suitable for normalizing gene expression in a novel model of Duchenne muscular dystrophy, the D2-mdx mouse
Source: PLoS One. 2024 Nov 13;19(11):e0310714. doi: 10.1371/journal.pone.0310714 (PMC11560031; doi:10.1371/journal.pone.0310714)
Supplement: S1 Table — (DOCX) [file pone.0310714.s007.docx]

|  | All data | Healthy | DMD | GC | DIA | Heart | Heart + | BL10 | mdx | DBA | D2mdx | 4wk | 8wk | 12wk | 28wk | 52wk |
| --- | --- | --- | --- | --- | --- | --- | --- | --- | --- | --- | --- | --- | --- | --- | --- | --- |
| V2/3 | 0.34 | 0.32 | 0.34 | 0.33 | 0.21 | 0.25 | 0.17 | 0.29 | 0.33 | 0.31 | 0.34 | 0.27 | 0.27 | 0.21 | 0.32 | 0.23 |
| V3/4 | 0.23 | 0.21 | 0.24 | 0.29 | 0.19 | 0.18 | 0.15 | 0.23 | 0.24 | 0.23 | 0.22 | 0.24 | 0.25 | 0.20 | 0.26 | 0.18 |
| V4/5 | 0.22 | 0.23 | 0.19 | 0.23 | 0.15 | 0.15 | 0.14 | 0.17 | 0.21 | 0.22 | 0.17 | 0.21 | 0.22 | 0.15 | 0.20 | 0.19 |
| V5/6 | 0.19 | 0.19 | 0.18 | 0.20 | 0.13 | 0.15 | 0.13 | 0.20 | 0.17 | 0.18 | 0.16 | 0.18 | 0.19 | 0.17 | 0.20 | 0.16 |
| V6/7 | 0.17 | 0.18 | 0.15 | 0.18 | 0.13 | 0.13 | 0.11 | 0.17 | 0.15 | 0.18 | 0.16 | 0.19 | 0.17 | 0.16 | 0.19 | 0.18 |
| V7/8 | 0.15 | 0.16 | 0.15 | 0.15 | 0.16 | 0.20 | 0.10 | 0.16 | 0.13 | 0.21 | 0.15 | 0.16 | 0.18 | 0.20 | 0.16 | 0.16 |
| V8/9 | N/A | N/A | N/A | N/A | N/A | N/A | 0.10 | N/A | N/A | N/A | N/A | N/A | N/A | N/A | N/A | N/A |
| V9/10 | N/A | N/A | N/A | N/A | N/A | N/A | 0.17 | N/A | N/A | N/A | N/A | N/A | N/A | N/A | N/A | N/A |
